# Access to essential technologies for safe childbirth: a survey of health workers in Africa and Asia

**DOI:** 10.1186/1471-2393-13-43

**Published:** 2013-02-20

**Authors:** Jonathan M Spector, Jonathan Reisman, Stuart Lipsitz, Priya Desai, Atul A Gawande

**Affiliations:** 1 Department of Health Policy and Management, Harvard School of Public Health, Boston, MA, USA; 2 Department of Internal Medicine-Pediatrics, Harvard - Massachusetts General Hospital, Boston, MA, USA; 3 Department of General Medicine, Brigham and Women’s Hospital, Boston, MA, USA

**Keywords:** Neonatal mortality, Maternal mortality, Childbirth, Developing world, Health technology

## Abstract

**Background:**

The reliable availability of health technologies, defined as equipment, medicines, and consumable supplies, is essential to ensure successful childbirth practices proven to prevent avoidable maternal and newborn mortality. The majority of global maternal and newborn deaths take place in Africa and Asia, yet few data exist that describe the availability of childbirth-related health technologies in these regions. We conducted a cross-sectional survey of health workers in Africa and Asia in order to profile the availability of health technologies considered to be essential to providing safe childbirth care.

**Methods:**

Health workers in Africa and Asia were surveyed using a web-based questionnaire. A list of essential childbirth-related health technologies was drawn from World Health Organization guidelines for preventing and managing complications associated with the major causes of maternal and newborn mortality globally. Demographic data describing each birth center were obtained and health workers reported on the availability of essential childbirth-related health technologies at their centers. Comparison analyses were conducted using Rao-Scott chi-square test statistics.

**Results:**

Health workers from 124 birth centers in 26 African and 15 Asian countries participated. All facilities exhibited gaps in the availability of essential childbirth-related health technologies. Availability was significantly reduced in birth centers that had lower birth volumes and those from lower income countries. On average across all centers, health workers reported the availability of 18 of 23 essential childbirth-related health technologies (79%; 95% CI, 74%, 84%). Low-volume facilities suffered severe shortages; on average, these centers reported reliable availability of 13 of 23 technologies (55%; 95% CI, 39%, 71%).

**Conclusions:**

Substantial gaps exist in the availability of essential childbirth-related health technologies across health sector levels in Africa and Asia. Strategies that facilitate reliable access to vital health technologies in these regions are an urgent priority.

## Background

Of the approximately 130 million births that take place each year, an estimated 3.3 million newborns die in the neonatal period [1], 1.2 million intrapartum-stillbirths occur [2], and nearly 350,000 mothers die from problems related to childbirth [3]. Most of these deaths occur in Africa and Asia and are entirely preventable [4-6]. The major causes of maternal and newborn mortality are well described. For women, these include postpartum hemorrhage, infection, obstructed labor, and hypertensive-related disorders; for babies, these are infection, intrapartum-related mortality, and complications related to prematurity [4,7-14]. In order to prevent childbirth-related deaths, skilled attendance at each and every childbirth has emerged to be a global priority. Women in high-risk regions are increasingly being encouraged to deliver in health facilities where potential complications can theoretically be better managed [15,16]. In practice, however, poor quality care at health facilities is frequently observed [17].

Guidelines published by the World Health Organization (WHO) and others identify standard practices proven to promote the safety of mothers and newborns around the time of childbirth [9-11,18]. Health technologies, defined as equipment, medicines, and consumable supplies, are crucial resources required by health workers to provide high quality care and successfully adhere to established childbirth guidelines [19]. Insufficient access to health technologies substantially impairs health workers’ ability to deliver minimum standards of care and, as a result, technology scarcity is recognized to be a major underlying cause of maternal and newborn deaths [20-22]. For example, poor availability of health technologies in smaller centers may compel patient referrals to higher level facilities. This risks unnecessary delay in care since many of these patients could have been better managed if appropriate technologies were otherwise present [23]. Most life-saving health technologies are not complex and costly but rather are simple and relatively inexpensive [19].

Few data exist that describe the general availability of childbirth-related health technologies in the parts of the world where rates of maternal and newborn mortality are greatest. The aim of this cross-sectional study was to develop an epidemiologic profile of the availability of “essential” childbirth-related technologies across a spectrum of health facilities in Africa and Asia. Essential health technologies are defined as those that are widely accepted to be required for provision of safe maternal and newborn care. Health workers in these regions were systematically surveyed to assess reliable access to essential childbirth-related health technologies.

## Methods

The childbirth-related health technologies assessed in this study were drawn directly from published WHO guidelines for managing complications associated with the major causes of maternal and neonatal mortality. Midwives, nurses, pediatricians, obstetricians, and medical officers that are members of the following organizations were e-mailed a request for participation with a corresponding link to the web-based survey: Global Alliance for Nursing and Midwifery listserv (approximately 2,500 members), numerous African and Asian midwife societies (approximately 300 members), CHILD2015 international child healthcare information and learning discussion group (approximately 3,000 members), and the American Academy of Pediatrics Section on International Child Health listserv (approximately 900 members). These organizations were selected based on their strong associations with birth facilities in Africa and Asia.

Inclusion criteria were clinicians that (a) currently work or have recently worked in birth facilities in Africa and Asia, and (b) had comprehensive knowledge of the childbirth-related health technologies available in the birth facilities in which they work or worked. Surveys were screened to ensure that only a single entry from each center was included in the analysis.

The web-based questionnaire was developed and designed using a secure, commercially available internet platform (SurveyMonkey; http://www.surveymonkey.com). This anonymous cross-sectional survey took place over a 10 month period, from June 2009, to March 2010. The questionnaire was comprised of sections focused on (i) birth facility demographic information including country location, annual birth rate, and childbirth team composition; (ii) the availability of general supplies and medicines including soap and clean water for handwashing, instruments (i.e. cannulae, needles, and syringes), thermometer, antibiotics, and oxygen; (iii) the availability of supplies and medicines that support maternal care including blood pressure cuff, urine dipstick, partograph, intravenous fluids, oxytocin, magnesium sulfate, anti-hypertensive medication, antenatal corticosteroids, and blood transfusion capacity; (iv) the capacity for cesarean section delivery on-site, capacity for referral for cesarean section, and average time of transport for women requiring referral for cesarean section; and (v) the availability of supplies and medicines that support newborn care including a sterile instrument for cutting the umbilical cord, clean towels for drying and cleaning the baby after birth, suction device, bag-and-mask, warming unit or incubator, phototherapy unit, vitamin K, and topical ophthalmic antibiotics. In all, availability of 23 essential technologies was assessed.

Data were imported from the SurveyMonkey website and downloaded directly into Microsoft Excel (Microsoft Corp, Redmond, WA). Countries were stratified by gross national income (GNI) per capita, which is the World Bank’s main criterion for classifying economies [24]. Characteristics of birth facilities were reported using percentages. Ninety-five percent confidence intervals were calculated for these percentages using generalized estimating equations, adjusting for clustering of birth facilities within countries [25]. Comparisons of characteristics across groups of birth facilities were performed using Rao-Scott chi-square tests [26], again adjusting for clustering of birth facilities within countries. All analyses were performed using SAS statistical software (*SAS Institute Inc 2010. SAS OnlineDoc, Version 9.2. Cary, NC. URLhttp://www.sas.com/*).

The study was declared exempt by the Office of Human Research Administration at the Harvard School of Public Health.

## Results

Surveys from 124 birth centers located in 41 countries were received and included in the analysis, including 26 African and 15 Asian countries (63% and 37%, respectively). No duplicate entries (i.e., multiple surveys from the same center) were received. The 41 countries represented three levels of national economy stratified by GNI per capita: 44% from low income economies, 39% from lower-middle income economies, and 17% from upper-middle income economies (Table 1). The centers, identified by respondents, were classified into three sizes based on annual birth volume: 12% of facilities were “low-volume,” averaging 100 births or less annually; 48% of facilities were “moderate-volume” averaging between 101 and 2,000 births annually; and 41% of facilities were “high-volume,” averaging greater than 2,000 births annually.

**Table 1:** Geographic location of survey participants

**Income level**	**Country**	**% (n)**	**0-100 births**	**101-2000 births**	**>2000 births**
Low (<$1,005)	Bangladesh	3.6 (2)	-	2	-
	Burkina Faso	1.8 (1)	-	1	-
	Central African Republic	1.8 (1)	1	-	-
	Chad	1.8 (1)	1	-	-
	Congo, Dem. Rep.	3.6 (2)	2	-	-
	Ethiopia	1.8 (1)	-	-	1
	Gambia	3.6 (2)	-	-	2
	Kenya	10.7 (6)	-	4	2
	Liberia	3.6 (2)	-	2	-
	Malawi	8.9 (5)	-	2	3
	Mozambique	1.8 (1)	-	1	-
	Nepal	5.4 (3)	2	1	-
	Niger	5.4 (3)	-	2	1
	Rwanda	5.4 (3)	-	2	1
	Sierra Leone	5.4 (3)	1	2	-
	Tanzania	10.7 (6)	1	1	4
	Uganda	16.1 (9)*	-	2	6
	Zimbabwe	8.9(5)	-	4	1
	*Subtotal*	*100(56)*	*8*	*26*	*21*
lower-middle ($1,006-$3,975)	Bhutan	2.3 (1*)	-	-	-
	Egypt	4.5 (2)	1	-	1
	Ghana	13.6 (6)	2	2	2
	India	20.5 (9)	-	6	3
	Indonesia	2.3 (1)	-	1	-
	Laos (Lao PDR)	6.8 (3)	-	1	2
	Mongolia	2.3 (1)	-	1	-
	Nigeria	9.0 (4)	-	3	1
	Pakistan	4.5 (2)	-	2	-
	Philippines	4.5 (2)	-	-	2
	Senegal	6.8 (3)	-	3	-
	Sudan	9.0 (4*)	-	3	-
	Swaziland	2.3 (1)	-	1	-
	Timor-Leste (East Timor)	2.3 (1)	-	-	1
	Vietnam	2.3 (1)	-	1	-
	Zambia	6.8 (3)	-	1	2
	*Subtotal*	*100(44)*	*3*	*25*	*14*
upper-middle ($3,976-12,275)	Botswana	12.5 (3*)	-	1	1
	China	25.0 (6)	2	1	3
	Jordan	4.2 (1)	-	-	1
	Malaysia	12.5 (3)	-	1	2
	South Africa	37.5 (9)	1	4	4
	Thailand	4.2 (1)	-	-	1
	Namibia	4.2 (1)	-	-	1
	*Subtotal*	*100(24)*	*3*	*7*	*13*
	*Total*	*100(124)*	*14*	*58*	*48*

*One survey response from each of the following countries did not disclose the number of annual births at that center: Uganda, Bhutan, Sudan, and Botswana.

The list of countries is stratified by gross national income (GNI) per capita.

The number of births shown corresponds to annual birth volume per facility.

**Figure 1:**
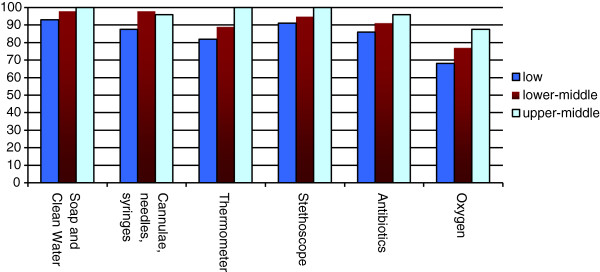
Availability of general essential technologies by national income level (GNI per capita).

**Figure 2:**
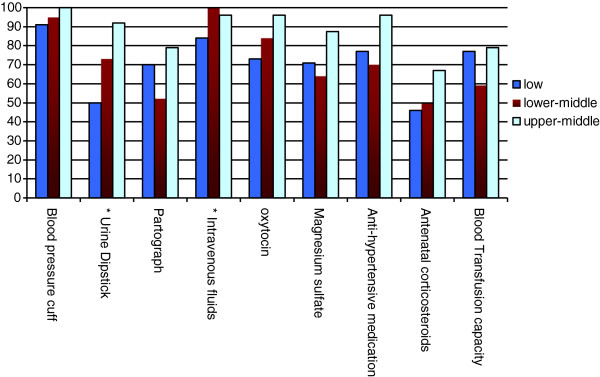
Availability of maternal health-related essential technologies by national income level (GNI per capita).

**Figure 3:**
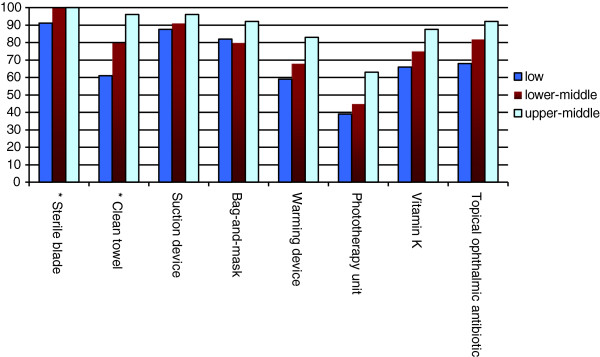
Availability of newborn health-related essential technologies by national income level (GNI per capita).

On average, health workers reported general availability of 18 out of 23 essential health technologies assessed (79%; 95% CI 74%–84%). Facilities from higher income countries were overall better equipped compared with facilities from lower income countries (Figures 1, 2, 3; p = 0.005). Health centers in low GNI countries reported reliable access to an average of 17 technologies (74%; 95% CI 66%–82%), centers in lower-middle GNI countries reported reliable access to an average of 18 technologies (79%; 95% CI 71%–87%), and centers in upper-middle GNI countries reported access to an average of 21 technologies (90%; 95% CI 84%–97%).

**Figure 4:**
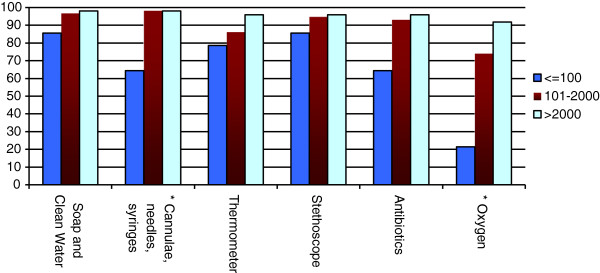
Availability of general essential technologies by annual birth volume per facility.

**Figure 5:**
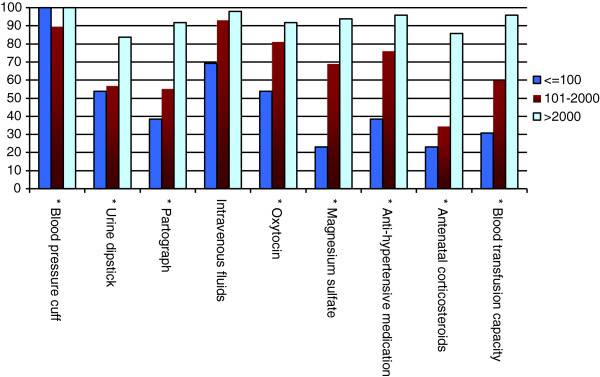
Availability of maternal health-related essential technologies by annual birth volume per facility.

**Figure 6:**
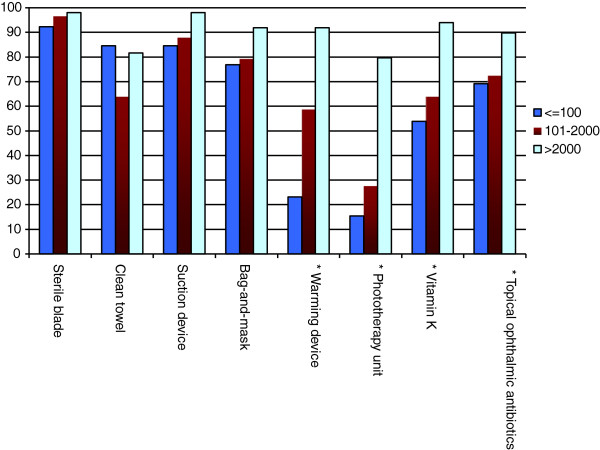
Availability of newborn health-related essential technologies by annual birth volume per facility.

The availability of essential technologies sorted by birth center volume is shown in Figures 4, 5, 6. Facilities with higher birth volumes had better access to most health technologies (p < 0.0001). On average, low-volume centers reported having reliable access to an average of only 13 of 23 technologies (55%; 95% CI 39%–71%), moderate-volume centers reported having reliable access to an average of 17 of 23 technologies (74%; 95% CI 68%–81%), and high-volume centers reported having reliable access to 21 out of 23 technologies (93%; 95% CI 88%–97%). Significant differences were found across centers of varying volume for the following technologies: instruments, oxygen, blood pressure cuff, urine dipstick, partograph, oxytocin, magnesium sulfate, anti-hypertensive medication, antenatal corticosteroids, blood transfusion capacity, warming unit or incubator, phototherapy unit, vitamin K, and topical ophthalmic antibiotics (all p < 0.05). There was a trend towards better availability of suction device at high-volume centers as compared to low-volume centers (p = 0.07).

The unavailability of essential childbirth-related health technologies was reported from virtually all centers. Among low-volume centers, more than 60% had poor access to a partograph, greater than 35% reported unavailability of antibiotics and oxytocin, and greater than 20% lacked thermometers and bag-and-masks. Among moderate-volume centers, 40% had unreliable access to a partograph and 35% lacked the capacity for blood transfusions. Even the highest volume centers had notable technology deficiencies; a blood pressure cuff was the only resource reliably available at 100% of the high-volume centers.

Across all facilities, midwives were reported to be the primary attendants at birth (overall present at 70% of births), followed by nurses (present at 36% of births), obstetricians (present at 32% of births), and pediatricians (present at 8.1% of births). Overall, cesarean section delivery was available at 73% of facilities. Centers with higher annual birth volumes were more likely to offer cesarean deliveries; 21% of low-volume centers, 66% of moderate-volume centers, and 96% of high-volume centers offered cesarean sections. All centers that did not offer cesarean deliveries on-site reported the capacity for maternal transport to a facility where cesarean section could be performed; however, maternal transport time was estimated at greater than two hours for 34% of facilities.

## Discussion

This cross-sectional survey and analyses of birth facilities in Africa and Asia demonstrated considerable gaps in the availability of essential equipment, medicines, and supplies necessary for the provision of safe childbirth care for mothers and newborns. Insufficient access to essential health technologies has been recognized as an underlying cause of poor quality of childbirth care and is linked with increased risks of adverse health outcomes [22]. For instance, medications such as uterotonics and magnesium sulfate must always be reliably accessible in order to prevent complications due to bleeding and hypertensive disease, respectively. Similarly, partographs or equivalent tools must be available to help diagnose prolonged and obstructed labor, and clean and functioning bag-and-mask devices must be immediately available in the event that newborn resuscitation is required. Approximately half of global deliveries take place in health care facilities and most maternal and newborn deaths are clustered around the time of delivery [4,27]. The results of this study support the premise that the accessibility of childbirth-related health technology plays a role in avoidable maternal and newborn harm.

Birth centers in lower income countries and those with lower birth volumes were overall less equipped. Birth centers in low volume centers were particularly resource-poor: reliable technologies were available only 52% of the time. While centers with larger birth volumes were overall better equipped, these birth facilities also fell short of universal access to essential equipment, medicines, and supplies.

This study provides a snapshot of general accessibility of essential childbirth-related health technologies in high priority countries. The limitations of the survey stem largely from the internet-based methodology that was utilized in order to assess a broad global population of maternal and newborn health workers. The survey instrument was distributed solely in English, which excluded non-English speakers from participating. Also, the response rate could not be calculated since, due to practical limitations imposed by the survey methodology, the number of clinicians invited to participate in the survey that met the relatively stringent inclusion criteria was not possible to determine. Participants required internet access to respond since the survey was electronically distributed. Internet technology is likely to be associated with respondents that work in more highly sophisticated birth centers and this is reflected in the relatively greater response rate from higher volume centers. However, this bias would generally be towards better equipped centers, suggesting that the technology gaps identified in this study may be an underestimate. Note that unsafe abortion is another major cause of maternal mortality globally but health technologies relating to this condition were not assessed since this study focused exclusively on equipment, medicines, and supplies required for safe childbirth.

## Conclusion

Substantial gaps exist in the availability of essential childbirth-related health technologies across health sector levels in Africa and Asia. Many high priority countries in these regions are suffering shortfalls in progress towards achieving Millennium Development Goals 4 and 5 [28,29]. Effective interventions that facilitate reliable access to essential childbirth-related health technologies are urgently needed in order to provide health workers with the tools they need to improve global maternal and newborn survival.

## Competing interest

The authors declare no interests, stocks, competing interests, or shares in organizations that may profit or lose through publication of this paper.

## Authors’ contributions

JMS, PD, and AAG were involved in conception and design of the study; JMS, JR, SL, PD, and AAG were involved in analysis and interpretation of data; JMS, JR, SL, PD, and AAG were involved in drafting and revising the paper. All authors read and approved the final manuscript.

## Pre-publication history

The pre-publication history for this paper can be accessed here:

http://www.biomedcentral.com/1471-2393/13/43/prepub
